# Characterization of absconding and attempts to abscond in a Colombian general hospital

**DOI:** 10.15649/cuidarte.4548

**Published:** 2025-10-14

**Authors:** Nicolas J Prada, Esteban Castrillón-Martínez, Ana María Perez-Gutierrez, Juan Pablo Zapata-Ospina

**Affiliations:** 1 Semillero de Investigación en Salud Mental y Psiquiatría (SISME), Facultad de Medicina, Universidad de Antioquia, Medellín, Colombia. E-mail: nicolas.prada@udea.edu.co Universidad de Antioquia Medellín Colombia nicolas.prada@udea.edu.co; 2 Área de Investigación, Hospital Alma Máter de Antioquia, Medellín, Colombia. E-mail: esteban.castrillonm@udea.edu.co Hospital Alma Máter de Antioquia Medellín Colombia esteban.castrillonm@udea.edu.co; 3 Servicio de Urgencias, Hospital Alma Máter de Antioquia, Medellín, Colombia. E-mail: ana.perez12@udea.edu.co Hospital Alma Máter de Antioquia Medellín Colombia ana.perez12@udea.edu.co; 4 Instituto de Investigaciones Médicas, Facultad de Medicina, Universidad de Antioquia, Medellín, Colombia. E-mail: juanp.zapata@udea.edu.co Universidad de Antioquia Medellín Colombia juanp.zapata@udea.edu.co

**Keywords:** Escape Reaction, Runaway Behavior, Patient Dropouts, Hospitalization, Reacción de Fuga, Conducta Fugitiva, Pacientes Desistentes del Tratamiento, Hospitalización, Reação de Fuga, Comportamento de Esquiva, Pacientes Desistentes do Tratamento, Hospitalização

## Abstract

**Introduction:**

Patient absconding in healthcare institutions has been associated with negative outcomes for both patients and the institutions themselves. Despite this, few studies have evaluated the frequency and characteristics of these events in Latin America, especially in general healthcare hospitals that do not specialize in psychiatric care.

**Objective:**

To describe the sociodemographic and clinical characteristics, as well as the event characteristics, among patients who absconded or attempted to abscond from a tertiary general hospital in Medellín, Colombia, between 2015 and 2023.

**Materials and Methods:**

Quantitative, descriptive cross-sectional study using secondary sources, where information was collected from the medical records of patients who absconded or attempted to abscond at the Hospital Alma Mater de Antioquia. A descriptive statistical analysis of the data was performed.

**Results:**

A total of 141 events were recorded during the evaluated period (135 absconds and 6 attempts to abscond). The period prevalence of absconding was 5.5 per 10,000 admissions, and most patients were young, single males, with a high frequency of substance use. Most events took place in the emergency department during the daytime.

**Discussion:**

Studying the epidemiological profile of absconding patients and their motivations could facilitate prevention and intervention.

**Conclusion:**

A lower absconding rate was found compared to reports from other countries. However, the characteristics of the patients were similar, with a predominance of young, single male patients with psychiatric history.

## Resumen

**Introducción:**

Las fugas de pacientes en instituciones de salud se han asociado con desenlaces negativos tanto para los pacientes como las mismas instituciones. A pesar de esto, pocos estudios han evaluado la frecuencia y características de estos eventos en Latinoamérica, especialmente en hospitales generales no especializados en atención psiquiátrica.

**Objetivo:**

Describir las características sociodemográficas, clínicas y de los eventos de pacientes que presentaron fuga o intento de fuga en un hospital de alta complejidad de Medellín, Colombia entre 2015 y 2023.

**Materiales y métodos:**

Estudio cuantitativo, descriptivo de corte transversal a partir de fuentes secundarias donde se recolectó la información de historias clínicas de pacientes que presentaron fuga o intento de fuga en el Hospital Alma Mater de Antioquia. Se realizó un análisis estadístico descriptivo de los datos.

**Resultados:**

Se presentaron 141 eventos durante el periodo evaluado (135 fugas y 6 intentos de fuga). La prevalencia de periodo de fuga fue 5,5 fugas por cada 10.000 ingresos y la mayoría de los pacientes fueron jóvenes de sexo masculino y solteros, con una alta frecuencia de consumo de tóxicos. La mayoría de los eventos ocurrieron en el servicio de Urgencias durante el día.

**Discusión:**

El estudio del perfil epidemiológico de los pacientes fugados podría facilitar su prevención e intervención.

**Conclusiones:**

Se encontró una tasa de fuga menor a las reportadas en otros países. A pesar de esto, las características de los pacientes fueron similares, predominando un patrón de pacientes masculinos, jóvenes, y solteros con antecedentes psiquiátricos.

## Resumo

**Introdução:**

Fugas de pacientes de instituições de saúde têm sido associadas a desfechos negativos tanto para os pacientes quanto para as próprias instituições. Apesar disso, poucos estudos avaliaram a frequência e as características desses eventos na América Latina, especialmente em hospitais gerais não especializados em atendimento psiquiátrico.

**Objetivo:**

Descrever as características sociodemográficas, clínicas e dos eventos de pacientes que sofreram fuga ou tentativa de fuga de um hospital de alta complexidade em Medellín, Colômbia, entre 2015 e 2023.

**Materiais e métodos:**

Foi realizado um estudo quantitativo, descritivo e transversal, utilizando fontes secundárias. As informações foram coletadas dos prontuários médicos de pacientes que sofreram fuga ou tentativa de fuga no Hospital Alma Mater, em Antioquia. Foi realizada uma análise estatística descritiva dos dados.

**Resultados:**

Houve 141 eventos durante o período de avaliação (135 fugas e 6 tentativas de fuga). A prevalência de períodos de evasão foi de 5,5 por 10.000 internações, e a maioria dos pacientes era jovem, solteiro, do sexo masculino e frequentemente envolvido no uso de substâncias. A maioria dos eventos ocorreu no Pronto-Socorro durante o dia.

**Discussão:**

Estudar o perfil epidemiológico de pacientesemevasãopodefacilitaraprevençãoeaintervenção.

**Conclusões:**

A taxa de evasão foi menor do que a relatada em outros países. Apesar disso, as características dos pacientes foram semelhantes, com predominância de pacientes jovens, solteiros, do sexo masculino e com histórico psiquiátrico.

## Introduction

Patient hospitalization is a medical decision made when an individual requires admission to a healthcare institution for a defined period in order to undergo diagnosis or treatment for a health condition. The hospitalization process concludes with hospital discharge, which may occur in various ways, including discharge due to clinical improvement as indicated by the attending physician, transfer to another facility, patient death, self-discharge, or the patient's absence without authorization from healthcare staff, a situation referred to as absconding^1^,^2^.

The definition of absconding varies considerably across studies, depending on the specified duration of absence and the institutional policies applied^1^,^3^,^4^. Due to these variations, significant differences in estimates of its frequency are observed. However, the main difference lies in whether the study was conducted in a psychiatric institution or in a general institution that is not specialized in this area. In psychiatric facilities, where most studies have been conducted, reported rates range from 0.2% to 54% of all admissions^5^, while in non-specialized institutions, rates vary from 0.27% to 2.4%^6^. With respect to the frequency of attempts to abscond, no specific data have been reported in the literature.

These types of events have been associated with various negative outcomes, most notably a considerable risk of suicide following absconding^7^, self-inflicted injury, and aggression toward others^1^ Consequently, they are considered a sentinel event for patient safety. It has also been observed that these patients require longer hospital stays and extended treatment, experience a loss of trust and engagement with healthcare services, and present increased use of psychoactive substances and other risk behaviors after the event^1^,^5^. From the institutional perspective, healthcare institutions have reported increased costs of care, risk of harm to other patients, property damages, and disruption of the work environment, all of which negatively impact patient care^1^.

Despite its relevance, studies on patient absconding and attempts to abscond remain scarce, with most research focusing on psychiatric institutions and overlooking non-specialized institutions where such events also occur^6^. In Latin America, the available evidence is even more limited, with only two studies conducted in Chile^8^,^9^, despite the fact that these phenomena are boldly influenced by contextual and regional factors, which may result in wide variations in frequency. In this context, there is a need to investigate these events in Colombia, particularly in a non-psychiatric institution with a high number of patients. The objective of this study was to describe the sociodemographic and clinical characteristics, as well as the event characteristics, among patients who absconded or attempted to abscond from a tertiary general hospital in Medellín, Colombia, between 2015 and 2023.

## Materials and Methods

A quantitative, descriptive cross-sectional study was conducted using secondary data sources.

**Participants and study setting**

Reports of absconding or attempts to abscond from the Alma Mater de Antioquia Hospital (HAMA) main headquarters, in Medellín, Colombia, between January 1, 2015, and August 31, 2023, were selected. During this period, 244,432 patients were admitted, among whom 141 incidents were documented (135 absconds and 6 attempts to abscond). Eligible participants were adults admitted to inpatient or observation services who were reported to have absconded or attempted to abscond during hospitalization. Patients escorted by correctional officers or those who did not authorize the use of personal data were excluded. For the purpose of this study, absconding was defined according to the HAMA institutional protocol as “the patient's departure from the medical care process without authorization, without a discharge form, and without the knowledge of healthcare staff.”^3^ An attempt to abscond was defined in accordance with the Protocol for Action in the Event of Patient Absconding of the Santiago Metropolitan Hospital as “surprising and stopping the act of a patient's absconding.”^4^

**Procedures**

For patient identification, reports of absconding and attempts to abscond during the study period were requested, which have been written according to the institutional protocol since 2015^3^. Each incident was recorded as a separate case if it occurred during a different hospitalization, since each episode had distinct characteristics. If both events occurred during the same hospitalization, only the final event (absconding) was recorded, and the sequence was noted in the study records.

**Variables**

Medical records were reviewed, and compliance with selection criteria was verified. Relevant sociodemographic variables were extracted (age, sex, marital status, type of residence, educational level, occupation, and type of health insurance). Clinical variables included history of mental disorder, suicide attempts, psychoactive substance use, and prior attempts to abscond during previous hospitalizations. Hospitalization- and event-related variables included patient's main admission diagnosis and the treating specialty, length of stay before the event, time of occurrence, type of hospital room, reports of restlessness, symptoms of craving, use of physical and/or pharmacological restraints, records indicating patient statements of intent to leave, as well as reports of self-inflicted injury, aggression toward others, or property damage during the event.

**Bias control**

The main bias was measurement bias associated with the study cross-sectional design that relied on secondary sources, as medical records may contain errors or missing information about the patients. To mitigate this, records were thoroughly reviewed, and the data recorded in the medical notes from different professionals (e.g., physicians, nurses) were cross-checked. Participants were selected based on the selection criteria, and all patients meeting these criteria were included.

**Statistical analysis**

Period prevalence was calculated by using the total number of hospital admissions per year during the study period, using the formula proposed by Molnar et al.^10^, which divides the number of events by the number of admissions. Descriptive statistics were applied to describe patient characteristics. For qualitative variables, absolute and relative frequencies were reported. For quantitative variables, means and standard deviations or medians and interquartile ranges were calculated, depending on whether the assumption of normality was met. Data were analyzed using R software in the RStudio environment, version 2024.04.1+748^11^. The dataset is available for free access and consultation in Mendeley Data^12^.

**Ethical considerations**

The study protocol was previously reviewed and approved by the HAMA Technical Research Committee (Code IN43-2023, Minutes No. 216) and complies with the principles of the Declaration of Helsinki and Colombian health research regulations.

## Results

During the study period, 141 events were documented, comprising 135 absconds and 6 attempts to abscond. A prevalence of 5.5 absconds per 10,000 admissions was found, while the annual prevalence varied between 3.5 and 9.1 absconds per 10,000 admissions across the study years. In the case of attempts to abscond, the overall prevalence was 0.26 per 10,000 admissions. The total and annual prevalence of both events are presented in Table 1.

**Table 1:** Annual and total point prevalence of absconding incidents and attempts to abscond in a tertiary general hospital in Medellín, Colombia

Year	Number of patients admitted	Absconding incidents and attempts to abscond	
		Number of events	Prevalence^1^
2015	28,298	14	4.9
2016	27,161	17	6.3
2017	27,623	25	9.1
2018	24,703	19	7.7
2019	26,070	10	3.8
2020	28,896	12	4.2
2021	31,356	15	4.8
2022	29,410	13	4.4
2023^2^	20,915	16	7.7
**Total**	**244,432**	**141**	**5.8**

^1^ Reported as the number of events per 10,000 admissions ^2^ Data includes patients admitted and incidents reported up to August 31, 2023.

**Sociodemographic characteristics**

Most patients who absconded were young males, single, and had some level of formal education. Occupation was unknown in the majority of cases, and most patients resided in urban areas, primarily in the municipality of Medellín, with others living in the Aburrá Valley. Patients who attempted to abscond were slightly older and all were male, while their remaining characteristics were similar to those patients who absconded. The complete sociodemographic characteristics are presented in Table 2.

**Clinical characteristics**

A history of absconding during previous hospitalizations was rare in both patient groups. However, a significant proportion of patients had at least one previously diagnosed mental disorder. While a history of substance use disorder was uncommon, more than half of the patients reported prior substance use (Table 3).

**Table 2:** Sociodemographic characteristics of patients who absconded and attempted to abscond from a tertiary general hospital in Medellín, Colombia. n=141

Characteristics	Absconded % (n=135)	Attempted to abscond % (n=6)	Total % (n=141)
Age in years			
Median (IQR)	36 [27 – 50]	40 [30 – 54]	36 [27 – 50]
Minimum – Maximum	18 – 85	21 – 63	18 – 85
Sex			
Male	65.90 (89)	100.00 (6)	67.40 (95)
Female	34.10 (46)	0.00 (0)	32.60 (46)
Marital status			
Single	68.80 (93)	50.00 (3)	68.10 (96)
Cohabiting	15.60 (21)	0.00 (0)	14.90 (21)
Married	11.90 (16)	16.70 (1)	12.10 (17)
Divorced	1.50 (2)	16.70 (1)	2.10 (3)
Unknown	2.20 (3)	16.70 (1)	2.80 (4)
Educational level			
No formal education	8.10 (11)	0.00 (0)	7.80 (11)
Elementary	34.10 (46)	16.70 (1)	33.30 (47)
High school	28.90 (39)	50.00 (3)	29.80 (42)
Higher education	13.30 (18)	33.30 (82)	14.20 (20)
Unknown	15.50 (21)	0.00 (0)	14.90 (21)
Occupation			
Homemaker	16.30 (22)	0.00 (0)	15.60 (22)
Unemployed	12.60 (17)	33.30 (2)	13.50 (19)
No occupation	8.20 (11)	0.00 (0)	7.80 (11)
Retired	5.20 (7)	0.00 (0)	5.00 (7)
Homeless	3.70 (5)	0.00 (0)	3.50 (5)
Other	24.40 (33)	33.30 (2)	24.80 (35)
Unknown	29.60 (40)	33.30 (2)	29.80 (42)
Type of residence			
Urban	89.70 (121)	100 (6)	90.10 (127)
Rural	4.40 (6)	0.00 (0)	4.30 (6)
Unknown	5.90 (8)	0.00 (0)	5.60 (8)
Health insurance			
Subsidized	54.10 (73)	33.30 (2)	53.20 (75)
Contributory	40.00 (54)	33.30 (2)	39.70 (56)
Special	5.90 (8)	33.30 (2)	7.10 (10)

Abbreviations: IQR = Interquartile range

**Table 3:** Clinical characteristics of patients who absconded or attempted to abscond from a tertiary general hospital in Medellín, Colombia. n=141

Characteristic	Absconded % (n=135)	Attempted to abscond % (n=6)	Total % (n=141)
History of absconding during previous hospitalizations			
Absent	94.80 (128)	83.30 (5)	94.30 (133)
Present	5.20 (7)	16.70 (1)	5.70 (8)
History of mental disorder			
No	60.70 (82)	33.30 (2)	59.60 (84)
Yes	39.30 (53)	66.70 (4)	40.40 (57)
Previous diagnosis of mental disorder*			
Bipolar disorder	20.00 (27)	0.00 (0)	19.20 (27)
Substance-related disorders	11.10 (15)	16.60 (1)	11.30 (16)
Schizophrenia	5.90 (8)	16.60 (1)	6.40 (9)
Depressive disorder	4.40 (6)	16.60 (1)	5.00 (7)
Anxiety disorder	2.90 (4)	16.60 (1)	3.50 (5)
Personality disorder	2.90 (4)	16.60 (1)	3.50 (5)
Other mental disorders	1.50 (2)	33.30 (2)	2.80 (4)
History of suicide attempts			
No	81.50 (110)	66.70 (4)	80.90 (114)
Yes	17.00 (23)	33.30 (2)	17.70 (25)
Unknown	1.50 (2)	0.00 (0)	1.40 (2)
History of toxic substance use			
No	40.70 (55)	16.60 (1)	39.70 (56)
Yes	57.10 (77)	83.40 (5)	58.20 (82)
Unknown	2.20 (3)	0.00 (0)	2.10 (3)
Type of toxic substance used*			
Marijuana	35.50 (48)	50.00 (3)	36.20 (51)
Cocaine and derivatives	35.50 (48)	33.30 (2)	35.50 (50)
Tobacco	29.60 (40)	33.30 (2)	29.80 (42)
Alcohol	21.50 (29)	33.30 (2)	21.90 (31)
Heroin	16.30 (22)	0.00 (0)	15.60 (22)
Benzodiazepines	4.40 (6)	0.00 (0)	4.30 (6)
Inhalants	3.70 (5)	0.00 (0)	3.50 (5)
Other stimulants	1.50 (2)	0.00 (0)	1.40 (2)

* Not mutually exclusive

**Event characteristics**

Most events occurred during the first days of hospitalization, primarily in the emergency department (Table 4). The majority of absconding incidents took place during the day shift, particularly in the late afternoon, with a peak between 5:00 pm and 7:00 pm. In contrast, most attempts to abscond occurred during the night shift. Reports of restlessness during hospitalization were relatively uncommon, as were reports of the need for physical or pharmacological restraint or the presence of symptoms of craving. Self-inflicted injury and aggression toward others were reported in only one case of attempted absconding, and no instances of property damage were documented.

**Table 4:** Characteristics of absconding events or attempts to abscond from a tertiary general hospital in Medellín, Colombia. n=141

Characteristics	Absconded % (n=135)	Attempted to abscond % (n=6)	Total % (n=141)
Days of hospital stay until the event			
Median (IQR)	2 [1 – 4]	5.5 [2 – 6]	2 [1 – 5]
Minimum – Maximum	0 – 94	1 – 7	0 – 94
Shift during which the event occurred			
Day (7 am – 7 pm)	68.10 (92)	33.30 (2)	66.70 (94)
Night (7 pm – 7 am)	31.90 (43)	66.70 (4)	33.30 (47)
Service where the event occurred			
Emergency	59.30 (80)	66.70 (4)	59.60 (84)
General inpatient service	40.70 (55)	33.30 (2)	40.40 (57)
ICU/SCU	0.00 (0)	0.00 (0)	0.00 (0)
Admissions diagnosis			
Mental and behavioral disorders	28.90 (39)	16.60 (1)	28.40 (40)
Trauma, poisoning, and other consequences of external causes	22.90 (31)	0.00 (0)	22.00 (31)
Symptoms, signs, and abnormal clinical and laboratory findings, not elsewhere classified	11.90 (16)	50.00 (3)	13.50 (19)
Circulatory system diseases	5.90 (8)	0.00 (0)	5.70 (8)
Respiratory system diseases	5.90 (8)	0.00 (0)	5.70 (8)
Digestive system diseases	4.40 (6)	16.60 (1)	4.90 (7)
Infectious and parasitic diseases	3.70 (5)	0.00 (0)	3.50 (5)
Other	16.30 (22)	16.60 (1)	16.30 (23)
Treating specialty			
General medicine	25.20 (34)	0.00 (0)	24.10 (34)
Internal medicine	20.70 (28)	33.30 (2)	21.30 (30)
Psychiatry	19.30 (26)	33.30 (2)	19.90 (28)
General surgery	8.10 (11)	16.60 (1)	8.50 (12)
Orthopedics	7.40 (10)	16.60 (1)	7.80 (11)
Plastic surgery	3.70 (5)	0.00 (0)	3.50 (5)
Other	15.60 (21)	0.00 (0)	14.90 (21)
Restlessness during hospitalization			
No	77.10 (104)	50.00 (3)	75.90 (107)
Yes	22.90 (31)	50.00 (3)	24.10 (34)
Need for pharmacological restraint			
No	78.50 (106)	50.00 (3)	77.30 (109)
Yes	21.50 (29)	50.00 (3)	22.70 (32)
Requirement for physical restraint			
No	87.40 (118)	50.00 (3)	85.80 (121)
Yes	12.60 (17)	50.00 (3)	14.20 (20)
Craving during hospitalization			
No	88.90 (120)	66.60 (4)	87.90 (124)
Yes	11.10 (15)	33.30 (2)	12.10 (17)
Prior statements of intent to leave			
No	72.60 (98)	50.00 (3)	71.60 (101)
Yes	27.40 (37)	50.00 (3)	28.40 (40)

Abbreviations: ICU=Intensive Care Unit. SCU=Special Care Unit. IQR= Interquartile range

**Patients diagnosed with mental and behavioral disorders upon admission**

Considering the differences in the frequency of events between psychiatric and non-psychiatric hospitals, a comparison was made between patients with and without a diagnosis of mental and behavioral disorders at the time of admission (Table 5). Although some characteristics of both groups were similar, patients who did have a diagnosis were younger, and few had a history of mental disorder. Similarly, they exhibited greater restlessness during hospitalization and required pharmacological and physical restraints.

**Table 5:** Characteristics of patients who experienced events according to the presence of a diagnosis of mental and behavioral disorders upon admission. n=141

Characteristic	Diagnosis of mental and behavioral disorder at admission		Total % (n=141)
	Yes % (n=40)	No % (n=101)	
Age in years			
Median (IQR)	32 [27 – 40]	38 [27 – 54]	36 [27 – 50]
Minimum – Maximum	18–63	18–85	18 – 85
History of mental disorder			
No	80.00 (32)	24.80 (25)	59.60 (84)
Yes	20.00 (8)	75.20 (76)	40.40 (57)
History of toxic substance use			
No	35.00 (14)	41.60 (42)	39.70 (56)
Yes	65.00 (26)	55.40 (56)	58.20 (82)
Unknown	0.00 (0)	3.00 (3)	2.10 (3)
Restlessness during hospitalization			
No	52.50 (21)	85.10 (86)	75.90 (107)
Yes	47.50 (19)	14.90 (15)	24.10 (34)
Need for pharmacological restraint			
No	52.50 (21)	87.10 (88)	77.30 (109)
Yes	47.50 (19)	12.90 (13)	22.70 (32)
Requirement for physical restraint			
No	70.00 (28)	92.10 (93)	85.80 (121)
Yes	30.00 (12)	7.90 (8)	14.20 (20)
Craving during hospitalization			
No	92.50 (37)	86.10 (87)	87.90 (124)
Yes	7.50 (3)	13.90 (14)	12.10 (17)
Service where the event occurred			
Emergency	97.50 (39)	45.60 (45)	59.60 (84)
General inpatient service	2.50 (1)	55.40 (56)	40.40 (57)

Abbreviations: IQR = Interquartile range

## Discussion

This study analyzed events of absconding and attempts to abscond from a tertiary general hospital in Medellín, Colombia, over a nine-year period and found a prevalence of 5.5 absconds and 0.26 attempts to abscond per 10,000 admissions. Most cases involved men in their forties, who were single and had an elementary or high school education. The prevalence observed in this study may be considered low compared with that reported in other non-psychiatric institutions: Iglesis et al.^9^ in Chile (0.4%), Anisi et al.^6^ in Iran (0.4%), Khammarnia et al.^13^ in Iran (0.5%), and Cheng et al.^14^ in Hong Kong (0.27%). Some authors suggested different reasons for these differences across contexts, highlighting the characteristics of health systems as a constant explanation for many of the causes of absconding^15^. It has been found that the inability to pay medical expenses and lack of insurance coverage are associated with vulnerable or migrant populations who abscond. Although the majority of patients included in this study had an unknown occupation, a low percentage of unemployed and homeless individuals were observed, in contrast to findings from other studies^13^. Furthermore, in this study, due to the provisions of the Colombian healthcare system, all patients had health insurance. In contrast, in other studies, the percentage of people without health insurance was over 50% in the groups of absconders^6^,^13^.

Other reasons that may contribute to absconding include the absence of standardized protocols and a lack of specific training for healthcare personnel in managing such situations^15^. Although the authors of the studies do not explicitly mention the existence of surveillance protocols in the hospitals examined, healthcare personnel have reported the need for clear and well-defined care guidelines to manage these events and mitigate their impact on patients and their environment^15^. Different institutional surveillance protocols, and even the lack of protocols in other contexts, may explain the differences in the frequency of events across studies, since absconding may be defined and managed differently across services, making comparisons difficult^16^.

In relation to patient characteristics, it was observed that absconders were predominantly young, male, and single, similar to findings in Chile by Valdivieso et al.^8^, and consistent with results from both psychiatric and non-psychiatric hospitals in different countries^14^,^17^,^18^. A history of substance use, reported in more than half of the study population, has also been identified as a risk factor for absconding in previous studies^6^,^18^. Yahyavi et al.^17^ reported bipolar affective disorder and substance use disorder as the most prevalent psychiatric diagnoses in their population, consistent with this study. In contrast, other research has identified schizophrenia as the predominant diagnosis^19^. Likewise, comparable lengths of stay prior to the event have been reported, with mental disorders frequently observed as the primary diagnosis and general medicine the most common treating specialty, both in general and psychiatric hospitals^14^.

Comparison of patients with and without a diagnosis of mental and behavioral disorders at admission revealed largely similar characteristics between the groups. However, a prior history of mental disorder was less frequent in the group diagnosed upon admission. This could be explained by the tendency for patients with an established history of mental illness who experience decompensation to be taken directly by relatives to specialized psychiatric centers. In contrast, those experiencing these symptoms for the first time are initially treated in general hospitals. As expected, patients diagnosed with mental and behavioral disorders exhibited greater restlessness and required more frequent use of pharmacological and physical restraints. Although craving was infrequently observed despite high rates of substance use, this may be explained by the anxiolytic effects of medications administered for restraint. Moreover, most of these patients were treated in the emergency department during initial acute management and/or pending transfer to mental health units, explaining why almost all events occur in this setting. This comparison by diagnostic category has not been performed in other studies in general hospitals, and it remains unclear whether similar patterns would be observed elsewhere.

Regarding the motivations behind absconding, Martin et al.^20^ proposed four categories to classify them. Absconding may be goal-oriented, such as the desire to use substances, significant life events, the need to protect other people or belongings, or the urgency to achieve or locate something specific. Given the high frequency of substance use in this study, it is possible that this was an important motivation in the cases observed. The presence of craving also raises the question of whether it would be necessary to implement intensive management of this symptom during the hospitalization of these patients.

Absconding may be associated with frustration with healthcare. This frustration may arise due to dissatisfaction with treatment regimens, physicians, staff, or length of hospital stay, as well as feelings of boredom, confinement, or fear. In this study, the length of stay was relatively short among patients; however, this concern could be addressed by providing a clear description of expected timelines, which would help reduce the uncertainty experienced by patients^21^. Symptomatic motivations for absconding generally involve patients who abscond due to episodes of psychosis or cognitive impairment^20^. In some of the cases studied, this could be related to diagnoses of schizophrenia, although no patients with formally diagnosed cognitive impairment who may have this motivation were found. Despite this, the possibility that other disorders or conditions leading to intense emotions, such as paranoia or fear, may have influenced the patients' decision to abscond cannot be completely ruled out. Finally, in some cases, absconding may occur due to the patient's impulsivity or the emergence of an opportunity, such as an unlocked door or window^20^. It is possible that the times when the highest number of absconds were observed, especially towards the end of the afternoon, are times when these events are more likely to occur. However, it should be noted that opportunities for absconding at the hospital where the study was conducted are limited by its infrastructure and the security measures implemented by the company responsible for the institution's security.

In other contexts, qualitative studies have been conducted to explore the motivations behind absconding in general hospitals^15^. Supplementing quantitative data with qualitative approaches would provide a more comprehensive understanding of the factors influencing these events, exploring patients' experiences and perceptions, as well as the barriers or facilitators perceived by healthcare staff. Furthermore, the use of mixed methods could offer a more detailed perspective, helping to identify patterns and design effective interventions. These approaches would not only allow the quantification of the prevalence of absconding but also enable the clarification of its underlying reasons and the development of prevention strategies more appropriate to hospital contexts.

Some strategies proposed in the literature to prevent absconding and attempts to abscond focus on improving communication between healthcare personnel and patients. Establishing closer relationships with patients and understanding their preferences and frustrations regarding hospitalization can facilitate person-centered care, which would help reduce potential conflicts and patients' disposition to abscond^15^. Providing clear and specific information about patients' health status and its practical implications may also reduce confusion and prevent absconding or attempts to abscond. There are non-restrictive interventions, such as the Safewards model^22^, which includes a set of simple interventions designed to make hospital units more person-centered. This model has been adopted and successfully evaluated in psychiatric hospitals worldwide^23^. Additional strategies to minimize harm and reduce the incidence of absconding include shared decision-making, close patient monitoring, careful communication of bad news to prevent conflict, and the use of assertive language to reframe potential areas of tension. Reducing the risk of absconding and harm to patients while they are hospitalized requires the implementation of a comprehensive approach that involves not only healthcare staff but also caregivers, family members, and patients themselves.

Other authors have suggested introducing structured activities to reduce boredom and frustration24 and adopting a recovery-oriented approach to promote less restrictive hospital environments, thereby reducing the incidence of absconding^24^,^25^. Identifying the characteristics of patients who abscond or attempt to abscond can not only improve prevention but also enable more active monitoring and the implementation of personalized alerts, which in turn could reduce the risk of complications and improve clinical outcomes. In this context, risk assessment tools such as absconding risk scales and risk profile identification could be highly valuable for conducting structured assessments of patients admitted and promoting prevention and surveillance strategies^26^-^28^.

Regarding attempts to abscond, during the review of medical records throughout the research, references to these events were identified in patients who did not have an official report of absconding attempts, suggesting that there may be significant underreporting of such incidents. This can be explained by the lack of explicit mention of such events in HAMA's institutional protocols^3^, indicating that a greater awareness of the characteristics of this event may be necessary. Nevertheless, the results regarding these patients remain relevant, as very few studies in the literature have specifically addressed this event^29^,^30^. A detailed study of their characteristics and associated motivations could play a key role in preventing absconding. One strategy to consider is designing research projects that use qualitative approaches to understand or explore the motivations of these patients. For example, techniques such as interviews allow for a deeper understanding of patients' direct experiences and motivations, as well as the economic, social, and psychological factors that influence their decision to abscond, something that clinical and sociodemographic data cannot always capture.

The limitations of this study include its descriptive design and the low number of reported attempts to abscond, which prevented making comparisons to identify differences between groups. The retrospective review of medical records limited the analysis to information that had been documented, leaving out unrecorded details. Likewise, due to the lack of information on the characteristics of all hospital admissions, it was not possible to calculate differential prevalence values according to specific variables, such as patients with and without a diagnosis of mental and behavioral disorders at admission. Nevertheless, to the best of our knowledge, this is the first study in Colombia to evaluate absconding and attempts to abscond among patients in general hospitals. It provides important groundwork for the development of new research aimed at identifying risk factors for absconding and attempts to abscond and proposing effective prevention strategies.

## Conclusions

A low prevalence of absconding was found compared with rates reported in the literature. Nevertheless, the characteristics of absconding patients were similar to those described in other countries, in both psychiatric and non-psychiatric hospitals, with most being young, male, and single. While a higher proportion of patients had a history of substance use, fewer had a history of mental disorder or were admitted with such a diagnosis. Identifying these characteristics may support the development of risk profiles for in-hospital surveillance and facilitate the prevention and management of these events.
